# Group Differences in Patterns of Retirement and Social Security Benefits Receipt in the United States

**DOI:** 10.1093/geroni/igaa057.367

**Published:** 2020-12-16

**Authors:** Hyesu Yeo

**Affiliations:** University of Georgia, Athens, Georgia, United States

## Abstract

Extended working lives are a clear phenomenon in the U.S. However, the Social Security benefits and old-fashioned retirement concept has not changed by the new trend. This study is to examine 1) whether retirement and Social Security benefits simultaneously happen as Social Security was planned, and 2) differences in groups based on the trajectories in retirement and Social Security benefits. This study used data from the Health and Retirement Survey (HRS) 2000-2016. The sample consisted of respondents the ages of 54 or older who worked for pay without receiving Social Security benefits in 2000 (N=1,924). A Latent Class Growth Analysis was conducted to classify the number of groups based on individuals’ trajectories in retirement and Social Security. The bivariate analysis tested group members’ characteristics. The final model included four groups: 1) ‘delayed retirement and Social Security,’ 2) ‘early Social Security but re-entry of the labor market after early retirement,’ 3) ‘delayed retirement but early Social Security,’ and 4) ‘retirement and Social Security at the same time point.’ The bivariate analysis demonstrated significant differences between groups in retirement age, Social Security age, education, health, and income. The result indicated that Group 2, with lower health status, education level, and income, was vulnerable in retirement. The group retired and received Social Security comparatively earlier, whereas they finally retired at the oldest age after reentering the labor market. This finding suggests that Social Security and retirement do not happen simultaneously, and Social Security is not enough for some people’s living.

